# Optimization of antimicrobial dosing in patients with acute kidney injury: a single-centre observational study

**DOI:** 10.1093/jacamr/dlac080

**Published:** 2022-07-25

**Authors:** Stephen Hughes, Katie L Heard, Nabeela Mughal, Luke S P Moore

**Affiliations:** Chelsea and Westminster NHS Foundation Trust, 369 Fulham Road, London SW10 9NH, UK; Chelsea and Westminster NHS Foundation Trust, 369 Fulham Road, London SW10 9NH, UK; Chelsea and Westminster NHS Foundation Trust, 369 Fulham Road, London SW10 9NH, UK; North West London Pathology, Imperial College Healthcare NHS Trust, Fulham Palace Road, London W6 8RF, UK; National Institute for Health Research Health Protection Research Unit in Healthcare Associated Infections and Antimicrobial Resistance, Imperial College London, Hammersmith Campus, Du Cane Road, London W12 0NN, UK; Chelsea and Westminster NHS Foundation Trust, 369 Fulham Road, London SW10 9NH, UK; North West London Pathology, Imperial College Healthcare NHS Trust, Fulham Palace Road, London W6 8RF, UK; National Institute for Health Research Health Protection Research Unit in Healthcare Associated Infections and Antimicrobial Resistance, Imperial College London, Hammersmith Campus, Du Cane Road, London W12 0NN, UK

## Abstract

**Background:**

Acute kidney injury (AKI) is a potential complication of systemic infection. Optimizing antimicrobial dosing in this dynamic state can be challenging with sub- or supra-therapeutic dosing risking treatment failure or toxicity, respectively. Locally, unadjusted renal dosing for the first 48 h of infection is recommended.

**Objectives:**

To determine the outcomes associated with this dosing strategy.

**Methods:**

A retrospective cohort analysis was undertaken in patients treated for Gram-negative bacteraemia with concurrent non-filtration dependent AKI from a single-centre NHS acute hospital (April 2016–March 2020). Patient demographics, microbiology data, antimicrobial treatment and patient outcome (in-hospital mortality and kidney function) were analysed.

**Results:**

In total, 647 episodes of Gram-negative bacteraemia (608 patients) were included; 305/608 (50.2%) were male with median age 71 years (range 18–100). AKI was present in 235/647 (36.3%); 78/647 (12.1%) and 45/647 (7.0%) having Kidney Disease Improving Global Outcomes-defined injury (stage 2) or failure (stage 3), respectively. In-hospital 30 day mortality was 25/352 (7.1%), 14/112 (12.5%), 26/123 (21.1%) and 11/60(18.3%) in patients with normal renal function, AKI stage 1, AKI stage ≥2 and established chronic kidney disease, respectively. Recovery of renal function at Day 21 or discharge was present in 105/106 surviving patients presenting with AKI stage ≥2. Time to recovery of AKI was similar in patients receiving full, low or no aminoglycoside (3 versus 4 versus 3 days, *P* = 0.612) and those receiving full- and low-dose β-lactam (3 versus 5 days, *P* = 0.077).

**Conclusions:**

There is a high burden of AKI in patients with Gram-negative bacteraemia. Dose adjustments of β-lactams may not be necessary in the first 48 h of infection-induced AKI and single-dose aminoglycosides may be considered for early empirical coverage.

## Introduction

Hospitalizations with systemic bacterial infections are common, perhaps increasingly so from an ageing population, increased patient comorbidities and changes in community antimicrobial use.^1^ Among admitted patients with haemodynamic compromise or sepsis, acute kidney injury (AKI; arising from including renal hypoperfusion, endothelial dysfunction and inflammation) can complicate management and increase infection-related morbidity and mortality.^2–4^ Early, appropriate antimicrobial therapy is an important intervention for improving patient outcomes^5,6^; yet identifying optimal antimicrobial choice and dose in the context of AKI is challenging. Prescribers must balance dose reduction (which may result in suboptimal treatment and further complication of infection) against full dosing (which may result in drug accumulation and resultant toxicity).^7^

There is growing evidence to support aggressive dosing of β-lactam antimicrobials in sepsis induced-AKI for the critical first 48 h of therapy.^8–10^ Increased volume of distribution (*V*) due to extracellular body water accumulation and concurrent fluid replacement during the initial phases of infection management can result in dilution of these hydrophilic therapies and sub-therapeutic levels.^11^ Similarly for aminoglycosides, dose reductions in response to AKI coupled with changing *V* results in suboptimal peak levels and reduce bactericidal activity.^12^ Single dose, or short-courses, with therapeutic drug monitoring, can mitigate the toxicity risks seen with prolonged courses.^13,14^ For fluoroquinolones, dose reductions are recommended to avoid accumulation, but the large *V* of this lipophilic group necessitates appropriate initial dosing to achieve satisfactory activity, meaning dose reductions for the first 48 h of AKI are not always necessary.^15,16^

To further complicate prescribing of antimicrobial dosing in AKI, estimates of renal function based on creatinine clearance calculations are validated in stable renal function only.^11,17,18^ Serum creatinine changes in dynamic AKI can be delayed by 24–36 h and can significantly over- or underestimate current function.^19^ Urine output may be a more accurate measure of current renal function in patients with AKI yet monitoring and interpretation can be challenging in practice.^11,20^ Dose adjustments to antimicrobials made by creatinine clearance estimates on serum creatinine in AKI are not reflective of current kidney function and are not advised. However, prescribers frequently dose reduce renally excreted antimicrobials in AKI reducing doses as extrapolated from chronic kidney disease (CKD) guideline recommendations. A survey of specialist pharmacists (*n *= 71) found dose reductions were advised in patients with presenting with sepsis-induced AKI for β-lactams (60%) and quinolones (21%) and dose reductions or avoidance of aminoglycoside recommended (73%).^7^

From 2015, the local institute’s antimicrobial prescribing guidelines have recommended the unadjusted renal dosing of some common antimicrobials for patients presenting with infection-associated non-filtration dependent AKI to address concerns about sub-therapeutic dosing in this critical phase. β-Lactams and quinolones, where indicated, should be unadjusted for AKI where baseline is normal or unknown for the first 48 h of therapy and reviewed thereafter for potential dose adjustments based on revised renal function. Single dose aminoglycosides (amikacin 15 mg/kg immediately and gentamicin 5 mg/kg immediately), based on actual or adjusted body weight in obese patients, are recommended for patients with new AKI where baseline renal function is normal or unknown. Repeat dosing is not routinely advised due to concerns about potential accumulation and increased toxicity of aminoglycosides; if required, pre-dose serum monitoring is required to demonstrate adequate clearance prior to subsequent administration. Aminoglycosides are commonly combined with β-lactam options for empirical management for Gram-negative infections to provide ESBL and AmpC extended activity as part of our antimicrobial stewardship strategy; carbapenems are reserved for initial therapy in patients with known resistant infections. Utilizing aminoglycosides until pathogen identification and susceptibility testing is part of the local antimicrobial stewardship strategy to spare carbapenem usage.

We undertook a retrospective observational cohort analysis in a large central London teaching hospital to identify the incidence of AKI in patients presenting with Gram-negative bacteraemias, the choice and initial dosing of empirical antimicrobials prescribed since introducing this change of practice, and renal recovery following AKI.

## Methods

### Study setting and design

A retrospective observational cohort analysis was undertaken of all hospitalized patients with microbiologically confirmed Gram-negative bacteraemia across a large single-centre NHS acute Trust; Chelsea & Westminster Hospital (London, UK). All adults (>18 years) with a confirmed Gram-negative bacteraemia on or during admission in the four fiscal years April 2016 to March 2020 were included. Patients with multiple blood culture results within 30 days of first isolate were deduplicated to a single (first) episode. Patients presenting on multiple episodes with positive blood cultures >30 days apart were analysed separately. Electronic patient records (Millennium^®^, Cerner Corp., USA, and ICNET^®^, Baxter, UK) and microbiology laboratory data (Sunquest^®^ v8.3) were interrogated to identify demographic details, clinical data and outcomes. Patient demographics (gender, age and renal function at baseline and throughout admission), microbiology data (including antimicrobial susceptibilities) and patient outcome [in-hospital mortality, length of stay and kidney function recovery (defined as recovery to baseline renal function)] were extracted.

### Definitions

AKI was defined using the Kidney Disease Improving Global Outcomes (KDIGO) classification system^21^ as an increase in serum creatinine by ≥1.5-fold, or ≥26.5 μmol/L within any 48 h period; time to recovery was measured as number of days from peak serum creatinine to reduction of 1.5-fold. AKI recovery was defined as resolution of serum creatinine to baseline or rapid improvement of renal function at time of discharge if earlier. Calculation of eGFR was achieved using the Modification in Diet and Renal Disease (MDRD) equation. CKD was defined using KDIGO classification.^22^ Urine output measurements were not routinely available. In-hospital mortality includes all-cause mortality.

Full-dose aminoglycosides were defined using local treatment guidelines as 15 mg/kg and 5 mg/kg for amikacin and gentamicin, respectively. Dosing is based on actual body weight unless obese where an adjusted body weight is recommended. Patients with renal dysfunction are commonly excluded from extended interval dosing aminoglycosides monographs (e.g. Urban or Hartford) and the use of multi-daily dose aminoglycosides (e.g. amikacin 7.5 mg/kg and gentamicin 2–3 mg/kg) at extended intervals of 24–72 h are advised. This is defined within the study as the low-dose regimen. Full-dose β-lactams, or quinolones where used, were defined as unadjusted licensed dosing as per national formulary (BNF).^23^ IV therapies were used; full doses include piperacillin/tazobactam 4.5 g three or four times a day IV, amoxicillin/clavulanate 1.2 g IV three times a day, cefuroxime 1.5 g IV three times a day, ceftriaxone 2 g IV once daily, meropenem 1–2 g three times a day, temocillin 2 g IV two to three times a day and amoxicillin 2 g IV three times a day; higher doses may be used for targeted site infections e.g. central nervous system. Low doses were defined as adjusted doses for reduced creatinine clearance as defined by the manufacturers’ datasheets within the UK.^23^

### Laboratory analysis

Blood cultures were investigated in line with the national UK Standards for Microbiology Investigations from PHE on the relevant media, atmospheres and duration noted in the relevant standard operating procedure.^24^ MALDI-TOF spectroscopy (Biotyper^®^, Bruker) was performed. Antimicrobial susceptibilities were determined by disc diffusion using EUCAST (v.9) criteria.^25^

Serum creatinine levels and eGFR were obtained over a 21 day period after bacteraemia identification. Baseline eGFR values were those taken on the bacteraemia confirmation date and were corroborated against previous measurements to ensure consistency. If this was absent or seemed anomalous to previous measures, the highest eGFR value post-AKI resolution was taken as baseline estimate. Antibacterial treatment prescribed within the first 72 h from blood culturing was analysed in patients with acute kidney injury or failure (defined as AKI stage 2 or above).

### Statistical analysis

Median and IQR were used to describe and compare age, eGFR, serum creatinine levels and length of stay post-bacteraemia. Univariate analysis on non-parametric data was performed using two-tailed Mann–Whitney *U*-test or Kruskal–Wallis test to evaluate continuous variables between groups and Wilcoxon test for comparing paired tests. Fisher’s exact test was used to evaluate categorical data. *P* values <0.05 were considered statistically significant, and OR and 95% CI were recorded. Data were recorded in Microsoft Excel^®^, and GraphPad Prism^®^ 8.1.1 software was used for univariate analysis and to generate graphical data.

### Ethical approval

All data were anonymized and analysed in Excel 2017. Ethics review and individual patient consent were waived for this retrospective cohort analysis undertaken by the infection team following review by the institution’s clinical governance department and was it was registered as a service evaluation. All data collected were stored in concordance with the Data Protection Act and the General Data Protection Regulation (GDPR) and anonymized as soon as practical to do so.

### Consent for publication

No data necessitating consent were used in this study.

## Results

### Patient cohort

A total of 647 episodes of Gram-negative bacteraemia in 608 individual patients were included in this study; 305/608 (50.2%) of patients were male and the median age was 71 years (range 18–100). Community-acquired or -onset bacteraemia, defined as less than 72 h from admission until culture, was identified in 545/647 (84%). In total, 72/647 (11.1%) of patient episodes were on a critical care ward at time of bacteraemia with a further 26/575 (4.5%) admitted to critical care within 7 days of bacteraemia. Enterobacterales were the most frequently isolated Gram-negative bacteria (591/647; 91.3%) with high rates of third-generation cephalosporin resistance (107/591; 18.1%) indicating the presence ESBL or AmpC enzymes (Table 1).

**Table 1.** Baseline patient characteristics for patients with AKI and non-AKI on presentation with new Gram-negative bacteraemia

	Non-AKI	AKI	*P* value
No. of patients	412	235	—
Age, years, median (range)	69 (18–100)	78 (25–98)	<0.001
Sex, male, *n* (%)	197 (47.8)	127 (54)	0.141
Community acquired/onset episodes, *n* (%)	343 (83.3)	202 (90)	0.432
Critical care admission during episode, *n* (%)	29 (7.0)	43 (18.3)	<0.001
Baseline GFR, mL/min, median (SD)	68.5(±22.7)	39.5(±18.1)	<0.001
Pathogen			
Enterobacterales, *n*	369	222	0.041
Gentamicin, % S	89.7	84.2	
Amoxicillin/clavulanate, % S	62.1	62.1	
3GC, % S	82.7	76.1	
Carbapenem, % S	100	99.5	
* Pseudomonas*, *n*	33	12	0.259
Gentamicin, % S	93.9	100	
3GC, % S	100	100	
Carbapenem, % S	81.8	100	
Other, *n*	10^a^	1^b^	

S, susceptible; 3GC, third generation cephalosporins.

a Other pathogens include *Achromobacter* spp. (2), *Campylobacter* spp. (2), *Moraxella* spp. (2) and *Neisseria* spp. (4).

b In the AKI group a single *Pasteurella* sp. was isolated.

Study-defined AKI was present in 235/647 (36.3%) of episodes, with 78/647 (12.1%) and 45/647 (7.0%) having KDIGO-defined injury or failure, respectively. CKD stage 4 or 5 was present in 54/647 (8.3%) of episodes and 6 cases had indeterminate staging of renal function due to insufficient sampling.

### Antimicrobial use

All antimicrobial prescribing in adult patients presenting for AKI (KDIGO stage) ≥2 was analysed (105/123) by the study team; 14 episodes were excluded due to treatment in critical care during contemporaneous haemofiltration, 2 cases were excluded due to transfer of patient to another trust within 24 h of bacteraemia and 2 patients did not receive antimicrobials due to palliation. Empirical aminoglycosides were prescribed in 87/105 cases (82.8%; amikacin 60 and gentamicin 27 prescriptions); median number of doses administered in the first 72 h was one. Full-dose aminoglycoside was used in 41/58 (70.7%) and 13/29 (44.8%) patients presenting with AKI stages 2 and 3, respectively (*P *= 0.0552). β-Lactam based therapy was prescribed in 97/105 cases with full licensed dosing (unadjusted for renal dysfunction) utilized in 86/97 cases (88.7%); 64/105 amoxicillin/clavulanate, 12/105 piperacillin/tazobactam, 9/105 cephalosporin, 7/105 carbapenem, temocillin 4/105 and 1/105 amoxicillin. Dose reductions of β-lactams were most common with amoxicillin/clavulanate dosing of 600 mg twice a day IV (*n *= 4) and 1200 mg twice a day (*n *= 5) with two dose-reduced courses of piperacillin/tazobactam (4.5 g twice a day IV). Five of the 11 dose-reduced β-lactams were adjusted to maximum dosing by the ward or specialist infection pharmacist within the first 24 h post bacteraemia. Fluoroquinolones (5/5), colistin (1/1), fosfomycin (1/1) and chloramphenicol (1/1) were dosed appropriately in the remainder of cases. In total, 104/105 patients received appropriate empirical therapy with at least one of the therapies providing targeted coverage to the respective pathogen.

### Impact of AKI on patients’ outcomes

**Figure 1.**
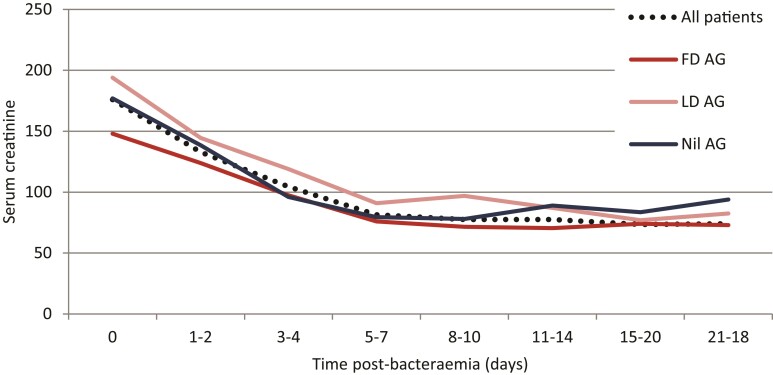
Median serum creatinine (μmol/L) recovery in patients presenting with Gram-negative bacteraemia and concurrent AKI stage ≥2; for all patients studied (*n *= 112) and also by aminoglycoside dosing received [FD AG, full dose aminoglycoside (5 and 15 mg/kg of gentamicin and amikacin, respectively), *n *= 54; LD AG, low dose aminoglycoside (2.5 and 7.5 mg/kg of gentamicin and amikacin, respectively), *n *= 33; or nil AG, no aminoglycoside treatment received, *n *= 20, as defined by the CWFT Adult Aminoglycoside Dosing guide (2021)].

The in-hospital 30 day mortality was higher in patients presenting with AKI stage ≥2 (26/123; 21.1%) than those with presenting with normal renal function (25/352; 7.1%; *P *= 0.0001) but similar to patients presenting with AKI stage 1 (14/112; 12.5%; *P *= 0.0802) (Table 2). Mortality for patients treated on critical care with concurrent AKI stage ≥2 was 5/14 (35.7%). Recovery of renal function at Day 21 or discharge was present in 105/106 surviving patients presenting with AKI stage ≥2 (Figure 1). Median time to recovery (1.5-fold reduction in serum creatinine) was 3 days (IQR 2–5). One patient presenting with AKI stage 2 had initial recovery of renal function after Day 5 but a second renal insult occurred with declining renal function after Day 15 consistent with an unresolved AKI. One case of unresolved AKI was present in the AKI stage 1 group (106/107). The median hospital length of stay was greater for patients presenting with AKI stage 1 and stage ≥2 compared with patients with preserved renal function (*P *< 0.0001).

**Table 2.** Crude patient-related outcomes (mortality, length of stay and recovered renal function at 30 days) at 30 days dependent on renal function on admission

	Normal renal function	AKI stage 1	AKI stage ≥2	CKD 4/5
Unadjusted 30 day in-hospital mortality, *n*/*N* (%)	25/352 (7.1)	14/112 (12.5)	26/123 (21.1)	11/60 (18.3)
Length of stay, days, median (range)	5.7 (0–307)	10.8 (0–195)	12.8 (1–122)	7.3 (0–78)
Recovery of renal function (at discharge or 21 days), *n*/*N*	—	100/101	96/97	—

### Impact of antimicrobial dosing on outcomes

Crude clinical recovery (alive and recovered AKI) at 30 days is reported for patients treated with β-lactam- and aminoglycoside-based therapy, at different degrees of study defined dosing. The use of an aminoglycoside as part of empirical therapy was not associated with improved patient outcomes [48/54 (89%) versus 27/33 (81.8%) with and without aminoglycoside therapy, respectively; *P *= 0.3189]; with no difference seen among patients receiving full- or low-dose aminoglycoside therapy [75/87 (86.2%) versus 15/20 (75%) with full and low dose, respectively; *P *= 0.3189]. Low-dose β-lactam therapy did not impact on clinical recovery [8/11 (72.7%) versus 73/86 (84.9%); *P *= 0.3831]; the small number of patients with a dose-reduced β-lactam limit this interpretation. Time to recovery of AKI was similar in patients receiving full, low or no aminoglycoside (3 days versus 4 days versus 3 days; *P *= 0.6183) and those receiving full and low-dose β-lactam (3 days versus 5 days; *P *= 0.0777).

## Discussion

This retrospective cohort analysis demonstrates the high burden of AKI in patients presenting with Gram-negative bacteraemia. Infection complicated with AKI is associated with patients with greater 30 day mortality and hospital length of stay.

In patients presenting with AKI stage ≥2 (injury or failure), early kidney function recovery was seen after 3 days in most patients. In patients surviving the acute bacterial illness, recovery of AKI (at Day 21 or discharge) was common as reported with previously published registry data.^26,27^ The insult to kidney function, in this small uncontrolled study, appears reversible upon targeted treatment of the infective cause.

Antimicrobial dosing within this study shows a high adherence to local guidelines recommending unadjusted antimicrobial dosing common within the first 48 h in patients presenting with renal dysfunction. Aminoglycosides, despite their association with nephrotoxicity when used in excess, were utilized as part of empirical treatment in the majority of patients presenting with Gram-negative bacteraemia with concurrent AKI. Full dosing of aminoglycosides for at least one dose in the first 48 h was common as part of initial empirical therapy despite AKI. The retrospective analysis is insufficient to link aminoglycoside use, and its dosing, with patient outcomes however no signal of harm is apparent from this data. Therapeutic drug monitoring is not advised for short-course (<48 h) therapy within our practice and a targeted β-lactam or quinolone is preferred from 48 h once identification and susceptibility of pathogen known. The truncated course length of aminoglycosides in this dynamic renal state may additionally reduce the nephrotoxic burden associated with aminoglycoside therapy.

Full-dose β-lactam therapy was commonly prescribed in patients with AKI stage ≥2 with no measured impact on long-term renal function noted. Dose reductions were uncommon and typically corrected by a pharmacist within 24 h to mitigate any potential sub-therapeutic dosing. Dose adjustments after 48 h were not recommended if renal function was improving to baseline at time of review to correct for any potential hyperfiltration with recovering function. Where renal function recovery was slow, dose adjustments based on the estimated renal function at 48 h were advised. Dose adjustments after 48 h were required to reduce further accumulation and minimize risk of β-lactam toxicity (e.g. neurotoxicity).

One of the biggest challenges is balancing the safety and efficacy of dosing of antimicrobials in this dynamic state. Therapeutic drug monitoring of antimicrobials would enable personalization of dosing in AKI however it is not routinely available for β-lactams and quinolones in our practice.^28^ Therefore, established dosing guidance in CKD is often extrapolated and applied to patients with new or acute kidney injury.^29^ Despite the well-established concept of sepsis-induced AKI, there is a paucity of published data to support the most appropriate antibacterial dosing option for patients presenting with AKI. What data are available are skewed to patients requiring renal replacement therapy. In our study, only a minority of patients presenting with AKI required haemofiltration support with most patients managed in a non-critical care setting. Here we describe the feasibility of a more aggressive dosing strategy in this critical period of infection. Adjusting dosing for AKI in the absence of CKD may not be always needed.

Therapies such as aminoglycosides, with a known nephrotoxicity profile, are often avoided in AKI due to concerns about accumulation and additive nephrotoxicity. Aminoglycosides make for a useful empirical treatment option in patients with suspected and confirmed Gram-negative invasive infections, alone or in combination to broaden activity of first-line β-lactams.^5,30^ Locally, a 24–48 h course of aminoglycoside is combined with a β-lactam (amoxicillin/clavulanate or cephalosporin) to cover for possible ESBL or AmpC resistant mechanisms. One in five Enterobacterales isolated in this study had one of these resistance mechanisms identified whilst almost two in five had amoxicillin/clavulanate resistance *in vitro*.

The high use of aminoglycosides supports the antimicrobial stewardship strategy and limits the use of empirical broad spectrum β-lactams such as piperacillin/tazobactam and meropenem. Despite the acuity of presentation, only 11.4% and 6.7% of patients with AKI received empirical piperacillin/tazobactam and carbapenems, respectively. Therapy is promptly adjusted once the identification and susceptibilities are known but indiscriminate use of these last-line therapies is avoided by providing robust empirical coverage with aminoglycoside combination therapy.

The retrospective design of our study inevitably reduces control over multiple confounders and data collection. Non-antibacterial treatments of AKI were not evaluated, including fluid replacement and inotropic support, which will impact upon AKI recovery. Kidney function was assessed using baseline eGFR and ongoing trends in serum creatinine response; urine output was not available for monitoring. Non-renal related complications of antibacterials were not assessed. Antibacterial treatment was assessed for the first 48 h in line with study design; follow-on antibacterial prescribing, including choice of route and duration, was not studied and will impact on patient outcomes.

In summary, this analysis highlights the high rate of transient AKI in patients presenting with Gram-negative bacteraemia. Due to the predicted pharmacokinetic changes and reversibility of the kidney insult, unadjusted doses of common antibacterials may be considered for the initial 48 h to minimize risk of sub-therapeutic dosing in this vulnerable patient group. Prospective studies are required to demonstrate the impact of antibacterial dosing in patients with invasive bacterial infections and transient renal function.

## Data Availability

The datasets analysed during the current study and further details on gaining access to the intervention reported within this study are available from the first author (S.H., e-mail: stephen.hughes10@nhs.net) on reasonable request, as long as this meets local ethics and research governance criteria.

## Transparency declarations

L.S.P.M. has consulted for/received speaker fees from bioMérieux (2013–21), Eumedica (2016–21) DNAelectronics (2015–18), Dairy Crest (2017–18), Profile Pharma (2018), Umovis Labs (2020–21), Shionogi (2021), Pulmocide (2021), Sumitovant (2021) and Pfizer (2018–21) and received research grants from the National Institute for Health Research (2013–21), Leo Pharma (2016), LifeArc (2020–21) and the CW+ charity (2018–21). N.M. has consulted for Pfizer (2019–21) and received educational support from Eumedica (2015) and Baxter (2017). S.H. has consulted for/received speaker fees Shionogi (2021), BowMed (2021), Eumedica (2020), Kent Pharma (2021), Gilead (2021), Tillots (2022) and Pfizer (2020–21) and received research grants from the CW+ charity (2021–22). All other authors: none to declare.

## References

[1] Gharbi M , Drysdale JH, Lishman H et al Antibiotic management of urinary tract infection in elderly patients in primary care and its association with bloodstream infections and all cause mortality: population based cohort study. BMJ 2019; 364: l525. 30814048 10.1136/bmj.l525 PMC6391656

[2] Bagshaw SM , Lapinsky S, Dial S et al Acute kidney injury in septic shock: clinical outcomes and impact of duration of hypotension prior to initiation of antimicrobial therapy. Intensive Care Med 2009; 35: 871–81. 19066848 10.1007/s00134-008-1367-2

[3] Peerapornratana S , Manrique-Caballero CL, Gómez H et al Acute kidney injury from sepsis: current concepts, epidemiology, pathophysiology, prevention and treatment. Kidney Int 2019; 96: 1083–99. 31443997 10.1016/j.kint.2019.05.026 PMC6920048

[4] Bagshaw SM , George C, Bellomo R. Early acute kidney injury and sepsis: a multicentre evaluation. Crit Care 2008; 12: R47. 18402655 10.1186/cc6863 PMC2447598

[5] Marquet K , Liesenborgs A, Bergs J et al Incidence and outcome of inappropriate in-hospital empiric antibiotics for severe infection: a systematic review and meta-analysis. Crit Care 2015; 19: 63. 25888181 10.1186/s13054-015-0795-y PMC4358713

[6] Kumar A , Ellis P, Arabi Y et al Initiation of inappropriate antimicrobial therapy results in a fivefold reduction of survival in human septic shock. Chest 2009; 136: 1237–48. 19696123 10.1378/chest.09-0087

[7] Mahmood S , Hughes S. A national survey to determine current practice regarding antimicrobial dosing in patients with sepsis-induced acute kidney injury (AKI). Federation of Infection Societies (FIS) Conference, Newcastle UK, 2018. https://hartleytaylor-registration.co.uk/fis2018/038.pdf.

[8] Ulldemolins M , Roberts JA, Rello J et al The effects of hypoalbuminaemia on optimizing antibacterial dosing in critically ill patients. Clin Pharmacokinet 2011; 50: 99–110. 21142293 10.2165/11539220-000000000-00000

[9] González de Molina FJ , Ferrer R. Appropriate antibiotic dosing in severe sepsis and acute renal failure: factors to consider. Crit Care 2011; 15: 175. 21861865 10.1186/cc10298 PMC3387596

[10] Blot S , Lipman J, Roberts DM et al The influence of acute kidney injury on antimicrobial dosing in critically ill patients: are dose reductions always necessary? Diagn Microbiol Infect Dis 2014; 79: 77–84. 24602849 10.1016/j.diagmicrobio.2014.01.015

[11] Matzke GR , Aronoff GR, Atkinson AJ et al Drug dosing consideration in patients with acute and chronic kidney disease-a clinical update from Kidney Disease: Improving Global Outcomes (KDIGO). Kidney Int 2011; 80: 1122–37. 21918498 10.1038/ki.2011.322

[12] Taccone FS , Laterre P-F, Spapen H et al Revisiting the loading dose of amikacin for patients with severe sepsis and septic shock. Crit Care 2010; 14: R53. 20370907 10.1186/cc8945 PMC2887170

[13] Paterson DL , Robson JMB, Wagener MM. Risk factors for toxicity in elderly patients given aminoglycosides once daily. J Gen Intern Med 1998; 13: 735–9. 9824518 10.1046/j.1525-1497.1998.00224.x PMC1497032

[14] Picard W , Bazin F, Clouzeau B et al Propensity-based study of aminoglycoside nephrotoxicity in patients with severe sepsis or septic shock. Antimicrob Agents Chemother 2014; 58: 7468–74. 25288085 10.1128/AAC.03750-14 PMC4249539

[15] Eyler RF , Mueller BA; Medscape. Antibiotic dosing in critically ill patients with acute kidney injury. Nat Rev Nephrol 2011; 7: 226–35. 21343897 10.1038/nrneph.2011.12

[16] De Paepe P , Belpaire FM, Buylaert WA. Pharmacokinetic and pharmacodynamic considerations when treating patients with sepsis and septic shock. Clin Pharmacokinet 2002; 41: 1135–51. 12405864 10.2165/00003088-200241140-00002

[17] Bragadottir G , Redfors B, Ricksten SE. Assessing glomerular filtration rate (GFR) in critically ill patients with acute kidney injury—true GFR versus urinary creatinine clearance and estimating equations. Crit Care 2013; 17: R108. 23767877 10.1186/cc12777 PMC4056314

[18] Awdishu L , Connor A, Bouchard J et al Use of estimating equations for dosing antimicrobials in patients with acute kidney injury not receiving renal replacement therapy. J Clin Med 2018; 7: 211. 10.3390/jcm7080211 PMC6111623 30103503

[19] Ostermann M , Joannidis M. Acute kidney injury 2016: diagnosis and diagnostic workup. Crit Care 2016; 20: 299. 27670788 10.1186/s13054-016-1478-z PMC5037640

[20] Molitoris BA , Reilly ES. Quantifying glomerular filtration rates in acute kidney injury: a requirement for translational success. Semin Nephrol 2016; 36: 31–41. 27085733 10.1016/j.semnephrol.2016.01.008 PMC4834456

[21] Khwaja A . KDIGO Clinical practice guidelines for acute kidney injury. Nephron Clin Pract 2012; 120: c179–84. 22890468 10.1159/000339789

[22] Levin A , Stevens PE, Bilous RW et al Kidney disease: improving global outcomes (KDIGO) CKD work group. KDIGO 2012 Clinical practice guideline for the evaluation and management of chronic kidney disease. Kidney Int Suppl 2013; 3: 1–150.

[23] NICE . British National Formulary (BNF). https://bnf.nice.org.uk/.

[24] PHE . Standards for Microbiological Investigation. https://www.gov.uk/government/collections/standards-for-microbiology-investigations-smi.

[25] EUCAST . Breakpoint Tables for Interpretation of MICs and Zone Diameters. 2019. http://www.eucast.org/fileadmin/src/media/PDFs/EUCAST_files/Breakpoint_tables/v_9.0_Breakpoint_Tables.pdf.

[26] Peerapornratana S , Priyanka P, Wang S et al Sepsis-associated acute kidney disease. Kidney Int Reports 2020; 5: 839–50. 10.1016/j.ekir.2020.03.005 PMC7270721 32518866

[27] Gameiro J , Carreiro C, Fonseca JA et al Acute kidney disease and long-term outcomes in critically ill acute kidney injury patients with sepsis: a cohort analysis. Clin Kidney J 2021; 14: 1379–87. 33959267 10.1093/ckj/sfaa130 PMC8087131

[28] Roberts JA , Paul SK, Akova M et al DALI: defining antibiotic levels in intensive care unit patients: are current β-lactam antibiotic doses sufficient for critically ill patients? Clin Infect Dis 2014; 58: 1072–83. 24429437 10.1093/cid/ciu027

[29] Crass RL , Rodvold KA, Mueller BA et al Renal dosing of antibiotics: are we jumping the gun? Clin Infect Dis 2019; 68: 1596–602. 30219824 10.1093/cid/ciy790

[30] Timoteus Deelen JW , Rottier WC, Buiting AGM et al Short-course aminoglycosides as adjunctive empirical therapy in patients with Gram-negative bloodstream infection, a cohort study. Clin Microbiol Infect 2021; 27: 269–75. 32387438 10.1016/j.cmi.2020.04.041

